# Clinical SWIR and CP-OCT imaging of interproximal lesions

**DOI:** 10.1186/s12903-024-04637-4

**Published:** 2024-08-17

**Authors:** Yihua Zhu, Oanh Le, Joany Xue, Spencer Wycoff, Daniel Fried

**Affiliations:** University of California, San Francisco, CA 94143-0758 USA

**Keywords:** Dental caries, Caries detection, Optical coherence tomography, SWIR imaging, Interproximal lesions

## Abstract

**Background:**

Enamel is highly transparent at short wavelength infrared imaging (SWIR) wavelengths allowing the detection of dental decay without the need for ionizing radiation. The purpose of this study was to use SWIR imaging methods including cross polarization optical coherence tomography (CP-OCT), occlusal transillumination (SWIR-OT), proximal transillumination (SWIR-PT), and occlusal reflectance (SWIR-R) to image interproximal lesions in vivo and compare the sensitivity with radiography.

**Methods:**

Participants (*n* = 30) aged 18–80 each with a radiopositive interproximal lesion scheduled for restoration were enrolled in the study. Studies have shown that the opposing proximal surfaces across the contact will likely also have lesions. SWIR images were acquired of the adjoining teeth at each contact with an interproximal lesion scheduled for restoration. Lesion presence and depth were assessed on each side of the contact for radiography and each SWIR imaging method. Lesions on radiographs and in CP-OCT images were identified by a single examiner while lesions in SWIR images were identified by a contrast threshold via semi-automatic image segmentation.

**Results:**

All SWIR imaging methods had significantly higher sensitivity (*P* < 0.05) than radiographs for the detection of interproximal lesions on the teeth opposite those restored. CP-OCT and SWIR-R imaging methods had significantly higher sensitivity than the other methods. SWIR imaging methods showed significantly higher lesion contrast than radiography.

**Conclusions:**

SWIR imaging methods can be used to detect interproximal lesions on posterior teeth with higher diagnostic performance than radiographs. CP-OCT appears well suited as a potential gold standard for the detection of interproximal lesions and assessment of their severity in vivo.

## Background

In the US, dental decay continues to be the leading cause of tooth loss even though caries progression is potentially preventable and reversible if detected early [1, 2]. New approaches are needed to detect lesions earlier than radiographs that are better suited for monitoring lesions over time. Imaging methods at short wavelength infrared (SWIR) wavelengths from 1000-2300-nm are highly promising due to the higher transparency of dental enamel [3–8]. Lesions can be imaged using transillumination and reflectance from tooth occlusal, buccal and lingual surfaces [3, 6, 9–11]. Interproximal lesions, the lesions located at the proximal contact points in between teeth, can be imaged via all three imaging geometries: SWIR occlusal transillumination (SWIR-OT), SWIR proximal transillumination (SWIR-PT), and SWIR reflectance (SWIR-R) imaging. SWIR-OT and SWIR-R have been combined into a single probe (SWIR-OTR) and tested in vivo [12–14].

Early lesions on proximal surfaces can be difficult to detect with radiography due to lack of physical access and the challenge of identifying the initial, subtle mineral loss of these lesions [15, 16]. Lesions are not typically visible on radiographs until decalcification has exceeded 30% [15, 16], while SWIR imaging methods are diagnostic with only 5% decalcification [17]. Radiographs markedly underestimate the depth and severity of interproximal lesions and clinicians generally assume that lesions penetrate much deeper than indicated in radiographs [18–20]. The first clinical SWIR study was carried out in 2010, when it was demonstrated that interproximal lesions that appeared on radiographs could also be detected in vivo using SWIR-OT and SWIR-PT imaging at 1310-nm with similar sensitivity [6]. Two later clinical studies showed that SWIR imaging methods have higher sensitivity than radiographs for the detection of dental caries, however those studies were limited to premolars that were scheduled for extraction [9, 21] for orthodontic reasons. Currently the only clinical infrared dental imaging systems that are commercially available operate at shorter near-IR wavelengths at 830 and 780-nm [22–25] where the enamel is less transparent [10]. The contrast between sound and demineralized enamel is markedly higher at wavelengths beyond 1400-nm [26–28] and stains do not interfere at wavelengths longer than 1200-nm [27]. In the latest clinical SWIR imaging study SWIR-OT and SWIR-R were combined in a single imaging device, that allowed simultaneous transillumination and reflectance measurements, SWIR-OTR [21].

Optical coherence tomography (OCT) which uses SWIR light at 1300-nm can also provide high resolution cross-sectional images of interproximal lesions from the occlusal surface. Lesions can be seen at depths of more than 3-mm [29–31]. Cross polarization OCT (CP-OCT) offers important advantages for imaging dental caries by reducing the strong reflection from tooth surfaces and enhancing the contrast between sound and demineralized dental enamel (30–31).

In this SWIR imaging study, test subjects with at least one radiopositive interproximal lesion scheduled for restoration were recruited. This new clinical study includes more advanced lesions and other teeth in addition to premolars that are not subject to crowding. The adjacent proximal surface at the contact point must either have a radiopositive lesion that appears confined only to enamel or have no visible lesion. Studies have shown that interproximal lesions are likely present on both sides of the contact even if both are not visible in radiographs [32, 33]. The two teeth at each contact point were imaged using SWIR-OT, SWIR-PT, SWIR-R imaging, and CP-OCT. The lesion contrast and penetration depth were also estimated from each image. Intraoral color images were also taken before and after cavity preparation and any demineralization on the exposed surface opposite the preparation was noted. The purpose of this study was to show that SWIR imaging methods have superior sensitivity for the detection of interproximal lesions on posterior teeth.

## Methods

### Participant recruitment and procedures

Study participants (*n* = 30) aged 18 + were recruited from the UCSF General Dentistry Clinic by the study investigators (UCSF IRB#19-27656). Informed consent was obtained from all subjects and/or their legal guardians. Participants were required to have one radiopositive interproximal lesion scheduled for restoration. Radiographs and treatment planning were carried out prior to recruitment by students and staff of the clinic independent of study investigators. Color images of the teeth on both sides of the contact were acquired before and after restoration using a FocusDent MD740 (Vilnius, Lithuania) Intraoral camera. Before restoration SWIR video was acquired using SWIR-PT from buccal and lingual views and SWIR-OTR over the occlusal surface. CP-OCT was used to acquire a 3D image 6 × 6-mm and 7-mm deep centered on each contact. Radiographical contrast was calculated using (I_S_-I_L_)/I_S_ for each lesion area that was identified. I_L_ was measured as the mean intensity over the lesion area at each proximal contact and I_S_ was selected at a sound position either directly below or directly above I_L_ to ensure a similar enamel thickness. Lesions on radiographs and demineralization in color images of proximal surfaces were identified by a single clinical examiner.

### SWIR occlusal reflectance and transillumination (SWIR-OTR)

**Fig. 1:**
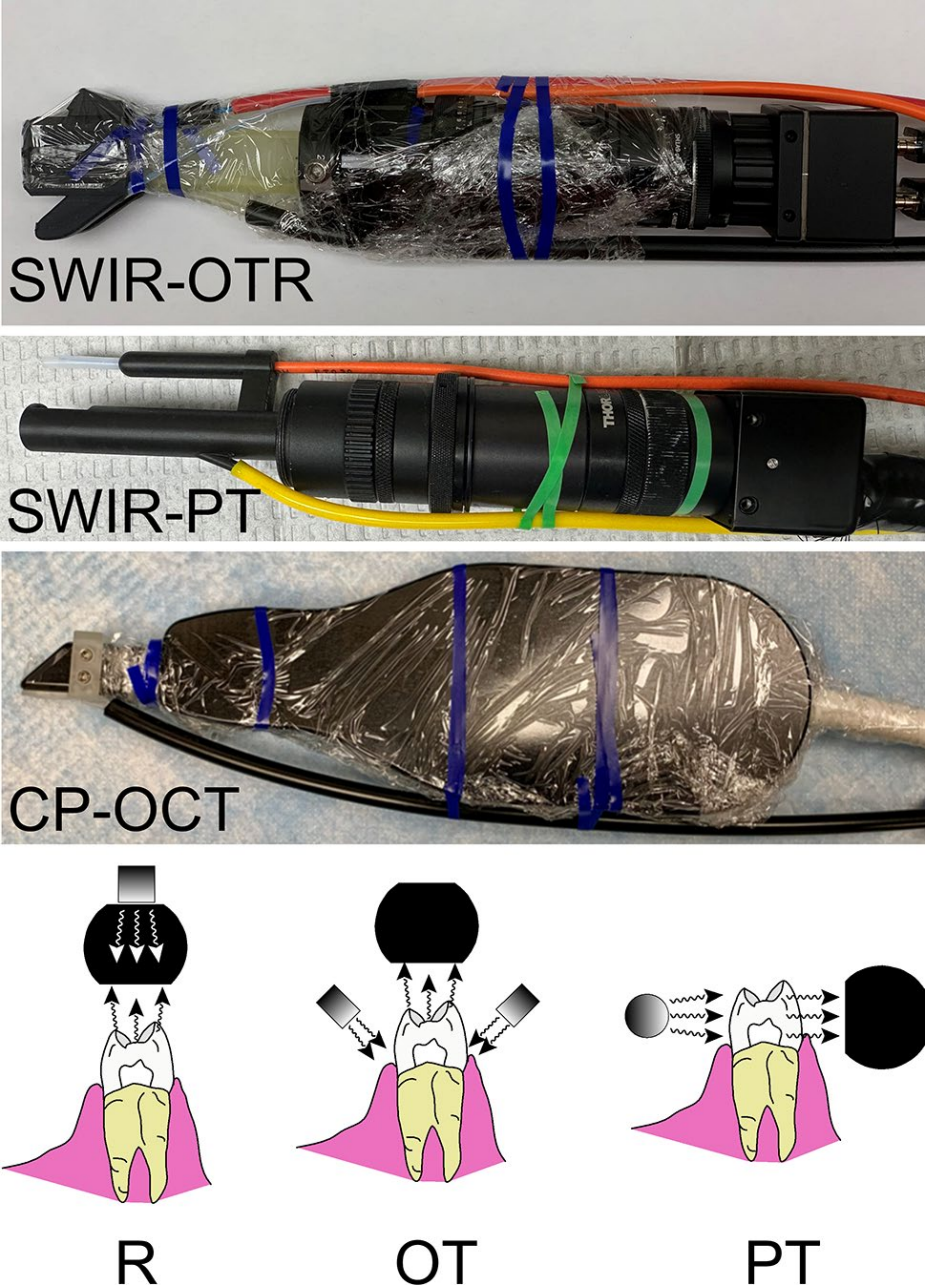
The three SWIR imaging devices used in this clinical study and the respective imaging geometries: Dual SWIR occlusal transillumination and reflectance (SWIR-OTR) clinical handpiece wrapped for infection control ready for clinical imaging. This device acquires both reflectance (R) and occlusal transillumination (OT) images from the occlusal surface. SWIR proximal transillumination (SWIR-PT) clinical handpiece that acquires proximal transillumination (PT) images from tooth buccal and lingual surfaces. Cross polarization optical coherence tomography (CP-OCT) handpiece wrapped for infection control that acquires images with a reflectance (R) geometry from tooth occlusal surfaces

Details regarding the fabrication of the dual occlusal transillumination and reflectance SWIR-OTR handpiece has been described previously [13, 14]. An image of the handpiece is shown in Fig. 1A. The SWIR images were captured using a Model SU640CSX (640 × 480 pixel) micro-SWIR focal plane array (FPA) from Sensors Unlimited (Princeton, NJ). The handpiece incorporated two superluminescent diodes operating at 1604-nm for reflectance and 1314-nm light for transillumination. The output intensity of each arm was set at 10-mW before entering the Teflon plugs located at the end of each arm. SWIR-R and SWIR-OT video was acquired at a rate of 30 Hz.

### SWIR proximal transillumination (SWIR-PT)

An image of the SWIR-PT handpiece is shown in Fig. 1B, it is similar to the device described previously [14]. Images were captured using another SU640CSX micro-SWIR FPA. Two planoconvex antireflection coated lenses of 75 and 100-mm focal length along with an adjustable aperture were placed between the handpiece and the InGaAs FPA to provide a field of view of 9 × 14-mm^2^ at the focus plane. The transillumination light is delivered through a 0.4-mm diameter low-OH optical fiber. A 1330-nm superluminescent diode (SLD) from Covega (Jessip, MD) with an output of 26-mW and a bandwidth of 36-nm was used as the light source for transillumination. The output intensity was set at 10-mW before entering the Teflon scattering rod. SWIR-PT video was acquired at a rate of 60 Hz.

### SWIR image processing and segmentation

**Fig. 2:**
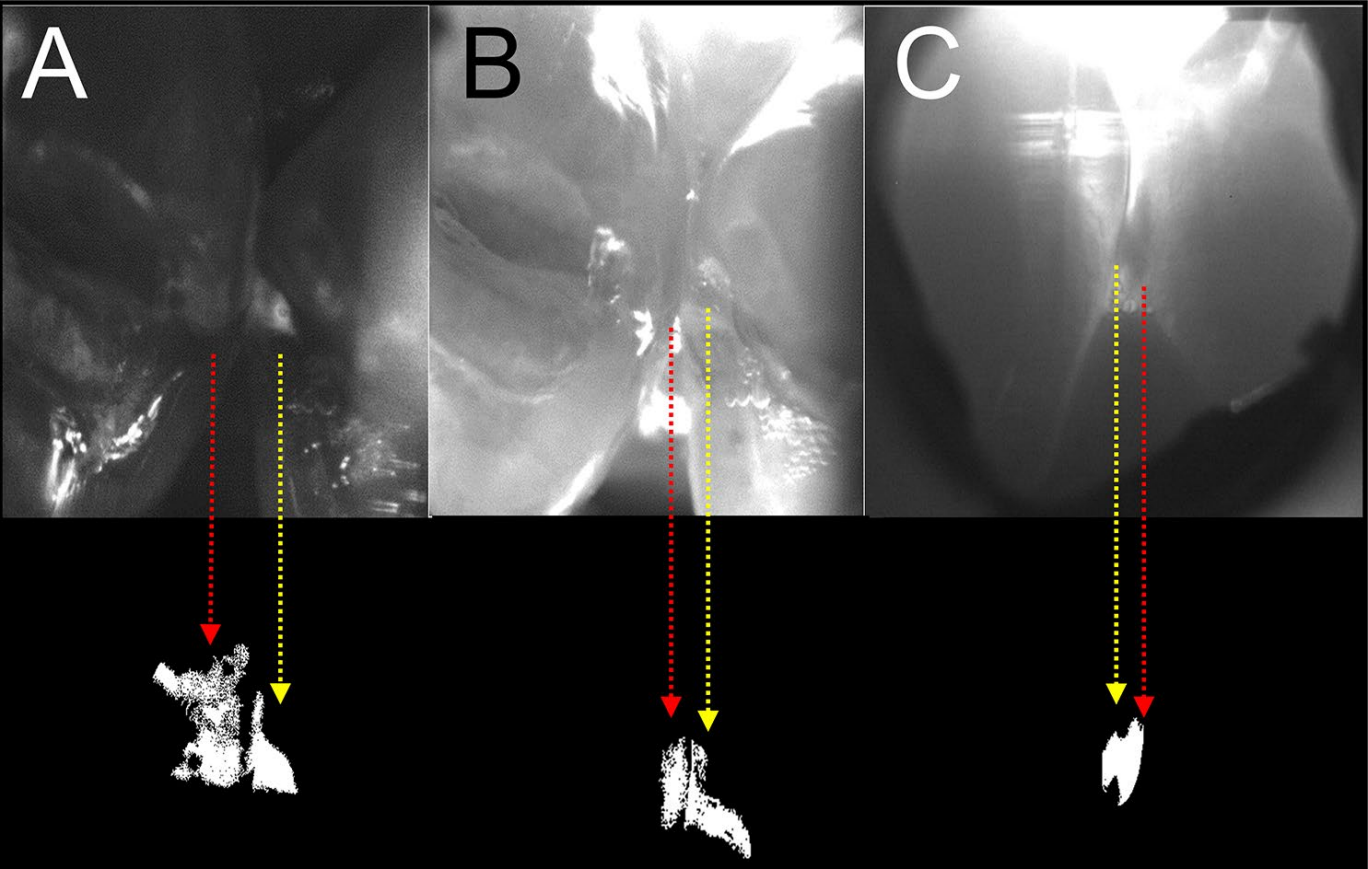
Segmented images showing extracted lesions areas from (**A**) SWIR reflectance (SWIR-R), (**B**) SWIR occlusal transillumination (SWIR-OT), and (**C**) SWIR proximal transillumination (SWIR-PT). Note these images match the images shown in Fig. 4E, F&G

Images were extracted from acquired videos and processed using MATLAB from Mathworks (Natick, MA). A semi-automatic image segmentation technique that was developed in a previous study was used to detect the interproximal lesions [21]. Raw SWIR images were first converted into contrast maps by applying the following transformation to every pixel in the image: (I_t_ – I_s_)/I_t_ for reflectance, and (I_s_ – I_t_)/I_s_ for transillumination. I_s_ was manually selected to the average intensity of sound enamel adjacent to the interproximal contact. I_t_ was the target pixel receiving the transformation. In a previous clinical study contrast was significantly higher (*P* < 0.05) between sound and interproximal lesion areas for SWIR imaging methods but not for radiography [21]. Therefore, contrast thresholds can be set for lesion detection for SWIR imaging methods but not for conventional radiography. A contrast of 0.1 is a reliable threshold for positive detection of interproximal lesions with reflectance and transillumination SWIR imaging. Pixels with contrast lower than 0.1 were removed to generate a binary mask showing every pixel with contrast higher than 0.1. Isolated areas of the mask adjoining the proximal contacts were designated as proximal lesion areas. This approach avoids high intensity areas caused by specular reflection (SWIR-R) or direct light that doesn’t pass through tooth structure (SWIR-PT and SWIR-OT). Specular reflection is easy to identify since it depends on angle of incidence and vanishes as the handpiece is rotated. Segmented lesions areas were isolated for depth and contrast measurements. Successful segmentation of an interproximal lesion with this technique was recorded as a positive detection. Failed segmentation of a lesion was considered a negative detection. If the detection was positive, lesion area (in pixels), mean contrast, and lesion depth were then automatically calculated following the semi-automatic lesion segmentation. Examples of lesion areas after segmentation are shown in Fig. 2 for SWIR-R, OT & PT.

### Cross polarization optical coherence tomography (CP-OCT)

The cross-polarization OCT system used for this study was the Model IVS-3000-CP from Santec (Komaki, Aichi, Japan). The swept-source system operates at a wavelength of 1321-nm with a bandwidth of 111-nm (11.4-µm axial-resolution) and can acquire complete tomographic images 6 × 6-mm with an axial depth of 7-mm in approximately 3-seconds. An image of the CP-OCT handpiece is shown in Fig. 1C. It has been used for multiple in vivo caries imaging studies and is described in more detail in those references [34–36]. An appliance made of autoclavable resin was placed on the distal end of the OCT scanning handpiece and the handpiece was covered with polyethylene film for infection control. Air at 10-psi was connected to the appliance to prevent fogging of the imaging window during image acquisition.

**Fig. 3:**
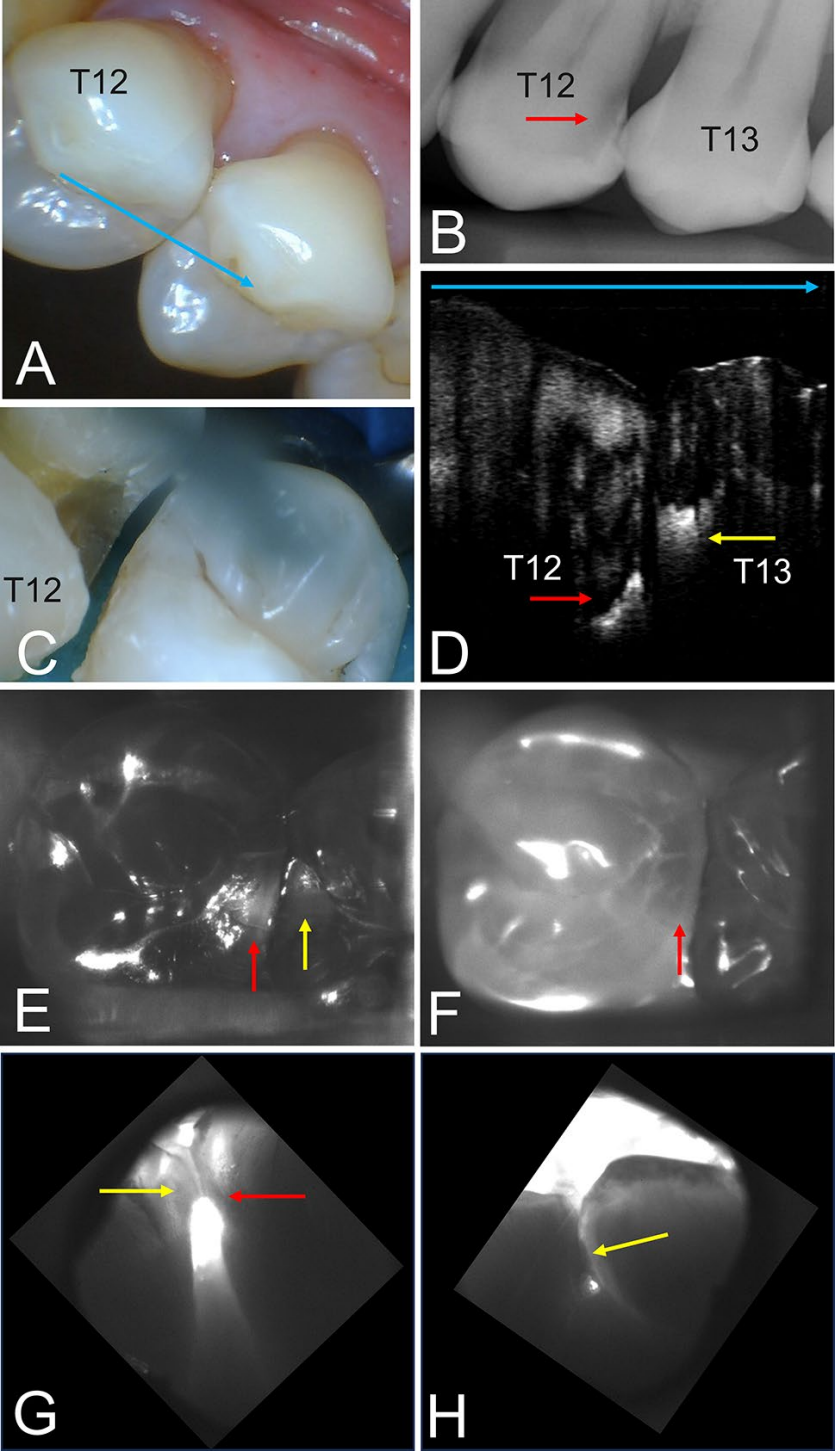
(**A**) Color image of the contact between tooth #12 (T12) and tooth #13 (T13), no lesions are visible. (**B**) Radiograph shows a large proximal lesion (red arrow) on tooth#12 at the distal contact that was scheduled for restoration. No lesion is visible on the opposing mesial contact of tooth #13. (**C**) After the cavity preparation decalcification is now visible on the mesial contact of tooth#13. (**D**) A CP-OCT b-scan image taken from the occlusal surface above the contact along the path of the blue arrow shown in (**A**) shows both proximal lesions that appear with increased subsurface reflectivity at the positions indicated by the red and yellow arrows. The CP-OCT scan shows the intensity with depth and lateral position with white high intensity and black low intensity. (**E**) SWIR-R occlusal reflectance shows lesions on both surfaces (red and yellow arrows) while (**F**) SWIR-OT only shows the lesion that was restored. (**G**) SWIR-PT image taken from the buccal side shows both proximal lesions across the contact while the (**H**) SWIR-PT image taken from the lingual shows only the lesion on tooth #13

**Fig. 4:**
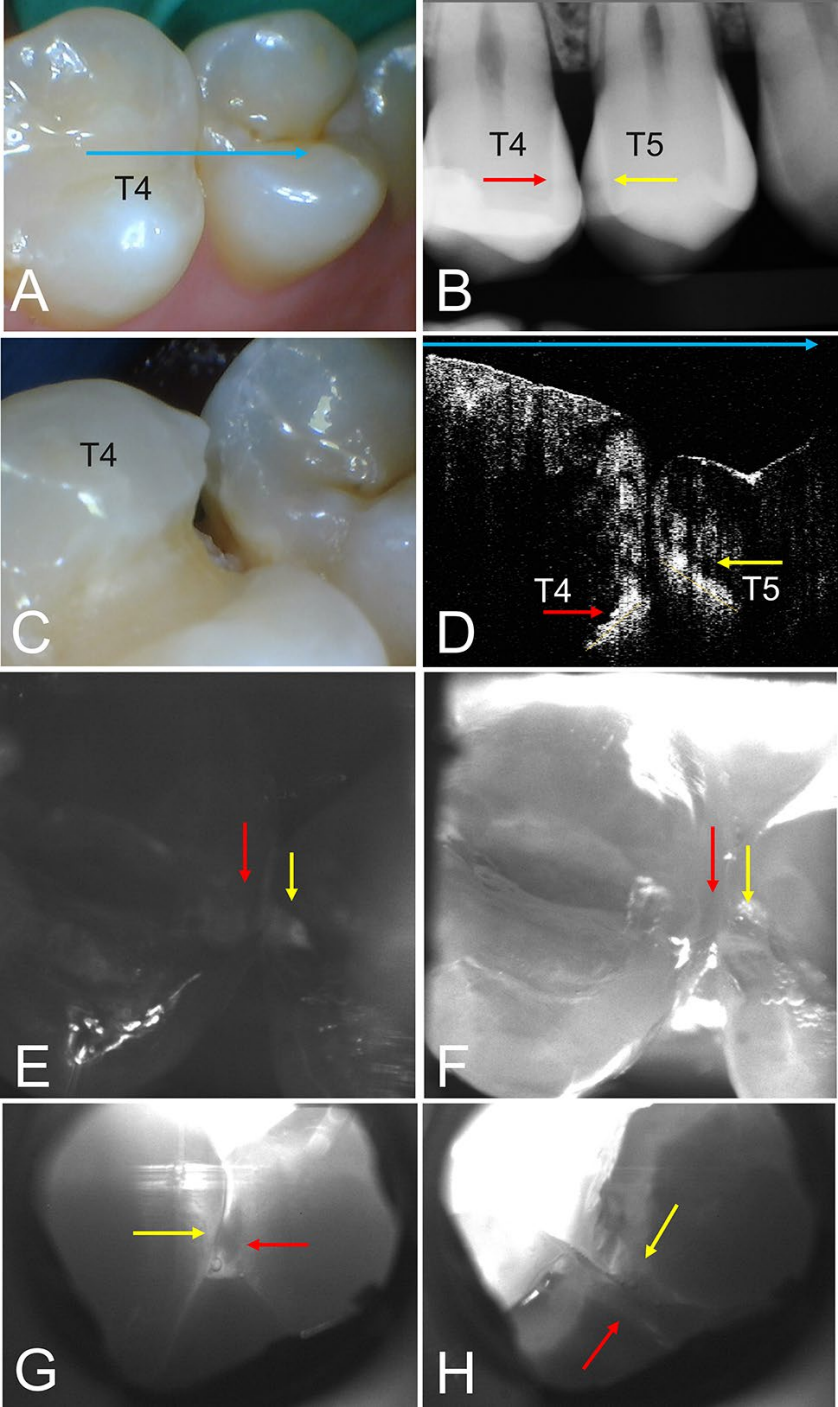
(**A**) Color image of the contact between tooth #4 (T4) and tooth #5 (T5), no lesions are visible. (**B**) Radiograph shows a lesion (red arrow) on tooth#4 at the distal contact that was scheduled for restoration and on the opposite contact on tooth#5 (yellow arrow). (**C**) After the cavity preparation decalcification is visible on the mesial contact of tooth#5. (**D**) A CP-OCT b-scan image taken from the occlusal surface above the contact along the path of the blue arrow shown in (**A**) shows both proximal lesions that appear with increased subsurface reflectivity at the positions indicated by the red and yellow arrows. The CP-OCT scan shows the intensity with depth and lateral position with white high intensity and black low intensity. (**E**) SWIR-R and (**F**) SWIR-OT images show lesions on both surfaces (red and yellow arrows). SWIR-PT images taken from the (**G**) buccal and (**H**) lingual sides show both proximal lesions across the contact

The reflected intensity in CP-OCT images decreases in sound enamel with increasing depth. Only increased reflectance from subsurface lesions or the dentinal-enamel junction (DEJ) can cause increased reflection. Only lesions on the proximal surfaces were imaged in this study and there was not interference from the DEJ. It is important to be aware of the intensity banding that can arise from the enamel birefringence and not confuse a maximum in one of the bands with a lesion. Fortunately, proximal lesions are sufficiently deep that the intensity maxima in the birefringence bands are of much lower intensity than the increased reflectance of the lesions. CP-OCT images of the proximal lesions in Figs. 3 and 4 both show banding due to enamel birefringence.

Acquired CP-OCT images from the Santec CP-OCT system were exported and further filtered with a median filter of kernel size 3 to reduce speckle noise and imported into Dragonfly from ORS software (Montreal, CA) for co-registration and analysis. Lesion depth was measured by applying a cylindrical ruler starting perpendicularly to the interproximal lesion surface. The scale of the ruler was customized to be the average intensity of the image inside a 100-µm disk surrounding the central axis of the ruler. Lesion depth was measured as the difference between the two lowest intensities along the ruler.

### Lesion detection rates, Lesion Contrast and lesion depth calculations

Radiography, CP-OCT, and visual inspection of opposing sites after restoration are all methods that have a high specificity for the detection of caries lesions. Therefore, the detection of any lesions by these methods was considered a true positive for the assessment of the lesion detection rates. A recent in vitro OCT study using *n* = 36 extracted teeth with 45 proximal lesions using histology as a gold standard showed that OCT had similar values of specificity and higher sensitivity and diagnostic accuracy to digital radiography [37]. According to these methods every surface was considered a positive lesion site for a total of *n* = 58 lesions.

Pixels were converted to mm for the SWIR and CP-OCT images for the lesion depth measurements by cross calibrating with a reference target. For the radiographs the mesial to distal distance in each radiograph was cross referenced with the corresponding mesial to distal distance in the SWIR images to convert pixels to mm.

Lesion detection rates were compared with radiography using Fishers’ exact test. Repeated measures ANOVA was used to compare the lesion contrast and lesion depth by removing all samples with missing values yielding a sample size of *n* = 26 for contrast and *n* = 24 for lesion depth. In addition, a mixed effects multiple comparisons model was employed that allows missing values. The results were the same for both methods. Prism statistical software from GraphPad Software, Inc., (La Jolla, CA) was used for the calculations. Significance level was set at *P* < 0.05.

## Results

Lesion detection rates are tabulated in Table 1 for all the lesions and lesions only located on the restored and opposing surfaces. The detection rates for all the lesions (*n* = 58), restored (*n* = 29) and opposing surfaces (*n* = 29) were all calculated as the fraction of the surfaces on which a lesion was detected for each method, and they are tabulated in Table 1. Even though half the lesion sites were selected based on their appearance on radiographs and scheduled for restoration, detection rates for the SWIR methods were all significantly higher than for radiography. SWIR-R and SWIR-PT were both significantly higher than other methods. Radiography detection rates for the surfaces opposing those restored provides a less biased comparison and the detection rates were much lower 0.38 versus 0.62 for all the lesions. In contrast the detection rates for the opposing surfaces with each of the SWIR methods was similar to the detection rates for all the lesions. All the SWIR detection rates were significantly higher than radiography. Also listed are mean lesion contrast values for radiography and SWIR-R, OT&PT and lesion depths for CP-OCT, and SWIR-R, OT&PT. The mean lesion contrast for the three SWIR methods SWIR-R, OT&PT was significantly higher than radiographs, nearly twice as high. Higher lesion depths were measured for OCT and SWIR-OT compared to radiography, but the difference was not significant. The lesion depths measured for SWIR-PT were significantly lower than for the other methods including radiography.

**Table 1:** The respective lesion detection rates for all the lesions and lesions only located on the restored and opposing surfaces. The mean(standard deviation) of the lesion contrast and the lesion depth for all detected lesions. Columns with the same letter in each row are statistically similar (*P* > 0.05)

	Radiograph	OCT	Visual (before)	Visual (after)	SWIR-*R*	SWIR-OT	SWIR-PT
**Detection Rate (All)**	0.62 ^a^	0.79 ^a, b^	0.05 ^c^	-	0.91 ^b^	0.74 ^a, b^	0.9 ^b^
Res Surface	1(0.93) ^a^	0.83 ^a^	0.10 ^c^	-	0.9 ^a, b^	0.76 ^b^	0.93 ^a, b^
Op Surface	0.38 ^a^	0.76 ^b^	0 ^c^	0.86 ^b^	0.93 ^b^	0.72 ^b^	0.86 ^b^
**Lesion** **Contrast**	0.13(0.064) ^a^	-	-	-	0.22(0.063) ^b^	0.20(0.010) ^b^	0.27(0.013) ^c^
**Lesion** **Depth (um)**	851(422) ^a^	1069(363) ^a^	-	-	830(379) ^a^	1049(514) ^a^	289(189) ^b^

Images acquired from the contact area in which the lesion on the opposing proximal surface was not visible in the radiograph are shown in Fig. 4. In Fig. 4B the radiograph shows a large lesion penetrating well into dentin on tooth#12 while there is no lesion visible on the opposing surface of tooth#13. The color image acquired after the restoration (Fig. 4C) shows some discoloration indicative of demineralization on tooth#13, but the severity of the lesion cannot be determined. The CP-OCT image in Fig. 4D shows strong reflections below the tooth surface at the position of the red and yellow arrows clearly indicating that lesions are present on both tooth#12 and tooth#13. The SWIR-R image shown in Fig. 4E acquired with the dual SWIR-OTR imaging probe shows lesions on both surfaces while SWIR-OT shows only one of the lesions. The SWIR-PT images acquired from buccal and lingual views are shown in Fig. 4G&H and the buccal view shows both lesions.

Images acquired from another contact area are shown in Fig. 3. For this example, lesions were identified in radiography on both sides of the contact. The color image acquired after the restoration (Fig. 3C) shows demineralization and staining on tooth#5. The CP-OCT image in Fig. 3D shows strong reflections below the tooth surface at the position of the red and yellow arrows clearly indicating that lesions are present on both tooth#4 and tooth#5. The SWIR-R and SWIR-OT images shown in Fig. 3E&F acquired with the dual SWIR-OTR imaging probe both show the lesions present on both surfaces. The SWIR-PT images acquired from buccal and lingual views are shown in Fig. 3G&H. Lesions are visible in both the buccal and lingual views.

## Discussion

In this study radiography and SWIR imaging methods were used to image the proximal contacts at which an interproximal lesion was scheduled for restoration. It is known that if a lesion develops on one side of the contact another lesion will likely also develop on the opposing tooth [32, 33] and lesions were identified on all of the opposing surfaces. No gold standards exist for the detection of caries lesions that can be used in vivo, however three of the methods, radiography, OCT, and visual examination of the exposed proximal surface after restoration have high specificity for caries detection and were used to identify surfaces as true positives. It is important to note that visual examination only shows that demineralization is present on the surface and gives no indication regarding the depth of lesion penetration, while both radiography and OCT show the depth penetration. Based on positives from any one of these three methods we found that all 58 of the proximal surfaces had lesions.

Comparison of lesion detection rates for the *n* = 29 surfaces opposite to those restored gives the best unbiased measure for lesion detection and all the SWIR methods were significantly higher than radiography with SWIR-R & PT having the highest performance. SWIR methods also yield much higher lesion contrast than radiography. High lesion contrast is particularly important for the implementation of auto-segmentation image analysis algorithms and machine learning. SWIR-OT & R and OCT provided similar lesion depth measurements to radiography which is important for lesion diagnosis. SWIR-PT provided high lesion contrast and high lesion detection rates. However, it greatly underestimated the lesion depths. In addition, imaging with SWIR-PT is more cumbersome than the other methods requiring two measurements per contact, buccal and lingual as opposed to a single measurement. Moreover, SWIR-PT can only be used as a stand-alone probe and is not useful for the detection of occlusal lesions. In contrast, SWIR-R & OT can be combined into a single multispectral SWIR-OTR probe that can be used for the detection of lesions on both proximal and occlusal surfaces and is more practical for caries screening.

CP-OCT performed extremely well for the detection of proximal lesions from tooth occlusal surfaces showing clear easily identifiable increases in intensity below the surface. Features in the images such as the subsurface rise in intensity and the reflectivity along the length of the lesion cannot be confused with false positives, nor can they be confused with increased reflectivity from the dentino-enamel junction (DEJ) due to the position and angle of the DEJ near tooth proximal surfaces. CP-OCT did miss a few of the lesions that appeared clearly on radiographs, however it appears that those lesions were located beyond the imaging range of the CP-OCT system. The scanning range of the system used was 7-mm in air which is greatly reduced by the high refractive index of enamel (1.6) to 4.4-mm. Other systems have been investigated in vitro with scanning ranges up to 10-mm that would be capable of reaching deeper lesions [37]. CP-OCT is a good candidate as a surrogate gold standard for interproximal lesions due to the potential high sensitivity and specificity. It is also well suited for automated image analysis and machine learning.

SWIR imaging devices operating beyond 1000-nm are not commercially available for dental imaging because of past restrictions due to military applications and high cost, however that is expected to change soon. In the past few years, costs have decreased markedly due to increased competition and production and there is a reduction in export restrictions for InGaAs devices. In addition, new SWIR imaging devices that use alternative semiconductor materials to InGaAs are under development for large imaging arrays using colloidal quantum dots that have improved sensitivity over InGaAs at longer SWIR wavelengths [38].

## Conclusions

SWIR imaging methods have significantly higher (*P* < 0.05) sensitivity for the detection of Class II interproximal lesions than radiographic and visual methods. CP-OCT appears well suited as a potential gold standard for the detection of interproximal lesions and assessment of their severity in vivo.

## Data Availability

The datasets used and/or analyzed during the current study are available from the corresponding author on reasonable request.

## Abbreviations

SWIR: Short wave infrared
NIR: Near infrared
CP: OCT-cross polarization optical coherence tomography
SWIR: OT-short wave infrared occlusal transillumination
SWIR: PT-short wave infrared proximal transillumination
SWIR: R-short wave infrared reflectance
SWIR: OTR-short wave infrared occlusal transillumination and reflectance
SLD: Superluminescent diode
FPA: Focal plane array
OCT: Optical coherence tomography
RM: ANOVA-repeated measures analysis of variance
DEJ: Dentino-enamel junction
